# Toll-Like Receptor-4 Mediated Inflammation Is Involved in the Cardiometabolic Alterations Induced by Intermittent Hypoxia

**DOI:** 10.1155/2015/620258

**Published:** 2015-03-19

**Authors:** Laureline Poulain, Vincent Richard, Patrick Lévy, Maurice Dematteis, Claire Arnaud

**Affiliations:** ^1^Université Grenoble Alpes, Laboratoire HP2, 38042 Grenoble, France; ^2^INSERM U1042, 38042 Grenoble, France; ^3^Université de Rouen, UFR Médecine-Pharmacie, 76183 Rouen, France; ^4^INSERM U1096, 76183 Rouen, France; ^5^CHU, Hôpital A. Michallon, Laboratoires du Sommeil et EFCR, 38043 Grenoble, France; ^6^CHU, Hôpital A. Michallon, Pôle Pluridisciplinaire de Médecine, 38043 Grenoble, France

## Abstract

*Objective*. Intermittent hypoxia (IH) is a major component of sleep apnea syndrome as its cardiometabolic complications have been mainly attributed to IH. The pathophysiology is still poorly understood but there are some similarities with the obesity-associated cardiometabolic complications. As the latter results from inflammation involving toll-like receptor-4 (TLR4) signaling, we assessed this pathway in the cardiometabolic consequences of IH. *Methods*. Lean adult male TLR4-deficient (TLR4^−/−^) mice and their controls (C57BL/6 mice) were exposed to either IH (FiO_2_ 21-5%, 1 min cycle, 8 h/day) or air (normoxic mice) for 4 weeks. Animals were assessed at 1-week exposure for insulin tolerance test and after 4-week exposure for morphological and inflammatory changes of the epididymal fat and thoracic aorta. *Results*. IH induced insulin resistance, morphological and inflammatory changes of the epididymal fat (smaller pads and adipocytes, higher release of TNF-*α* and IL-6) and aorta (larger intima-media thickness and higher NF*κ*B-p50 activity). All these alterations were prevented by TLR4 deletion. *Conclusion*. IH induces metabolic and vascular alterations that involve TLR4 mediated inflammation. These results confirm the important role of inflammation in the cardiometabolic consequences of IH and suggest that targeting TLR4/NF*κ*B pathway could represent a further therapeutic option for sleep apnea patients.

## 1. Introduction

Obstructive sleep apnea (OSA) is a public-health problem as it affects at least 10% of the middle aged men and represents a main cause of cardiovascular morbidity and mortality [1]. An independent association between OSA, insulin resistance, and type 2 diabetes has been consistently demonstrated by a number of cross-sectional, observational, and large population-based studies [2, 3]. Moreover, OSA patients have increased carotid intima-media thickness (IMT), an early sign of atherosclerosis, which correlates with nocturnal oxygen desaturation, independently of other cardiovascular risk factors [4, 5]. OSA severity may also predict occult coronary atherosclerosis in healthy overweight or obese male subjects [6]. Repetitive upper airway collapses during sleep result in intermittent hypoxia (IH) which is thought to be responsible for OSA-associated cardiometabolic complications, including atherosclerosis and insulin resistance [7]. Data obtained from animals exposed to IH, a validated experimental model of sleep apnea, showed that activation of the sympathetic nervous system and systemic inflammation underlie IH-induced metabolic and vascular consequences [7–9]. We also recently demonstrated that IH-induced inflammatory changes of epididymal white adipose tissue (EWAT) contributed to these outcomes [10].

In obesity, it is increasingly recognized that chronic activation of inflammatory signaling pathways is causally linked to insulin resistance [11] and vascular alterations [12]. Recent studies suggest that these deleterious effects could be mediated, at least in part, through the activation of toll-like receptors (TLR), and in particular the TLR4. TLRs are a family of pattern-recognition receptors that play a critical role in the innate immune system by activating proinflammatory signaling pathways in response to microbial pathogens [13]. Lipopolysaccharide (LPS) binds to TLR4, triggering a downstream cascade, which leads to the activation of the proinflammatory nuclear factor kappa-B (NF*κ*B) pathway and finally to the expression of numerous proinflammatory molecules, such as interleukin (IL)-6 and tumor necrosis factor (TNF)-*α* [14]. Furthermore, studies on different strains of mice showed that expression and activation of TLR4 are involved in aortic inflammation [15] and atherogenesis [16]. Together these experimental data confirm the major role of TLR4/NF*κ*B pathway in the crosstalk between inflammation, atherosclerosis, and metabolism dysfunctions.

In C57BL/6 mice, we previously found that IH-induced cardiovascular inflammation was characterized by an increased activity of NF*κ*B in aortic [17] and cardiac tissues (unpublished data). We also reported that, in lean animals, EWAT exposed to IH became pathological, behaving like excess fat in obesity, as it exhibited increases in macrophage recruitment and secretion of IL-6 and TNF-*α* [10]. Collectively, these data suggest that cardiometabolic complications due to IH and obesity may share some pathophysiological mechanisms. Therefore, we assessed whether metabolic and vascular consequences induced by IH involved the proinflammatory TLR4/NF*κ*B pathway activation.

## 2. Methods

### 2.1. Animals

Male TLR4-deficient mice (TLR4^−/−^, C57BL/6 background) were developed initially by Dr. Shizuo Akira (Research Institute for Microbial Diseases, Osaka, Japan) and were obtained from the EMMA (European Mouse Mutant Archive) network in Orleans, France. Seventeen-week-old male TLR4^−/−^ mice and their control groups (C57BL/6 mice) fed on a standard-chow diet were used. They were weighed throughout the experiments. The study was conducted in accordance with the European Convention for the Protection of Vertebrate Animals Used for Experimental and Other Scientific Purposes (Council of Europe, European Treaties ETS 123, Strasbourg, 18 March 1986) and with the* Guide for Care and Use of Laboratory Animals* (NIH Publication no. 85-23, revised 1996).

### 2.2. Intermittent Hypoxia

TLR4^−/−^ and their control C57BL/6 mice were divided into 2 subgroups, exposed to either intermittent hypoxia (IH) or normoxia (N). IH was performed as previously described [10]. The four groups of animals were exposed to the IH stimulus during daytime (*n* = 10 per cage, 8 h/day, cyclic 21-5% FiO_2_, 60 s cycle (60 events/h), lowest blood oxygen saturation up to 60%) for 4 weeks. FiO_2_ was measured with a gas analyzer (ML206, ADInstruments) throughout the experiment. Control animals (normoxic mice, N) were exposed to air in similar cages to reproduce similar noise and turbulences to those of the IH stimulus. Ambient temperature was maintained at 20–22°C.

During the first week of IH exposure, intraperitoneal insulin tolerance test (IpITT) was performed to assess global insulin sensitivity and, on the day following the last exposure period, fasted animals were sacrificed under anesthesia with intraperitoneal injection of ketamine (100 mg·kg^−1^) and xylazine (10 mg·kg^−1^) for further analysis.

### 2.3. Intraperitoneal Insulin Tolerance Test (IpITT)

Mice were fasted for 5 hours and then weighted before blood was collected from the tail tip for baseline glucose determination (*t* = 0). Blood glucose was measured using the OneTouch Ultra glucometer. Insulin (0.5 IU·kg^−1^ body weight, Novo Nordisk A/S, Bagsvaerd, Denmark) was injected intraperitoneally, followed by further blood glucose measurements at 15, 30, 60, and 90 minutes after the injection. The lowest blood glucose level (nadir) following insulin administration was calculated for each experimental group.

### 2.4. Blood Cholesterol Measurements

At the time of sacrifice, blood was collected by cardiac puncture on EDTA tubes. The plasma fraction was collected after blood centrifugation during 10 minutes at 11000 rpm (4°C). Total cholesterol was measured in plasma by a colorimetric enzymatic reaction using the Infinity kit (ThermoElectron Corporation, Massachusetts, USA) according to the manufacturer's guidelines.

### 2.5. Epididymal White Adipose Tissue (EWAT) Alterations

Bilateral epididymal fat pads were collected, weighted, and either fixed in 90% ethanol for adipocyte morphology study or incubated for cytokine determinations.

#### 2.5.1. Adipocyte Morphology

Ethanol-fixed, paraffin-embedded EWAT were sectioned (3.5 *μ*m), deparaffinized in toluene and rehydrated in descending ethanol series, and then stained with hematoxylin-eosin to assess tissue morphology. Adipocyte size was measured from photographs (10 × 40 magnification) using the NIS-Elements microscope imaging software (Nikon).

#### 2.5.2. Cytokine Secretion

Each EWAT pad was divided into two equal pieces and incubated at 37°C with mild shaking in RPMI medium. After 120 minutes of incubation, IL-6 and TNF-*α* were measured in the supernatants using an ELISA test according to manufacturer's instructions (R&D System Europe, Lille, France). Cytokine concentrations were expressed as ng/mL for 1 g of adipose tissue.

### 2.6. Assessment of Vascular Inflammation and Remodeling

#### 2.6.1. Aortic Intima-Media Thickness (IMT)

IH and N aortas were embedded in optimum cutting temperature (OCT) compounds (Tissue-Tek, Sakura Finetek Europe BV, Alphen aan den Rijn, The Netherlands), sectioned (10 *μ*m), and stained. Hematoxylin-eosin staining was used to assess the intima-media thickness (IMT). Morphometric analysis (up to 15 measurements on 10 noncontiguous midthoracic descending aorta sections per animal) was performed with a light microscope (Nikon Eclipse 80i, Nikon) and the NIS-Elements microscope imaging software (Nikon Instruments Europe BV).

#### 2.6.2. NF*κ*B Activity

We investigated whether IH could activate NF*κ*B by assessing the expression of its activated subunit NF*κ*B-p50 which has translocated into the nucleus. Nuclear NF*κ*B-p50 was determined in thoracic aorta of mice exposed to N or IH. Tissue homogenization and proteins extraction were performed according to manufacturer's instructions using a nuclear extract kit (Active Motif Europe, Rixensart, Belgium). Proteins concentration was evaluated using the BCA assay (ThermoScientific, Massachusetts, USA). Nuclear proteins were assayed for the presence of the activated p50 by ELISA using the TransAM NF*κ*B-p65/p50/p52 kit (Active Motif Europe). NF*κ*B activity was expressed in arbitrary units.

### 2.7. Statistical Analysis

Results were expressed as mean ± standard errors of the means (SEM) and analyzed using 2-way ANOVA and subsequent Bonferroni's multiple post hoc comparisons or Mann-Whitney* U* test. Statistical significance was set at *P* < 0.05.

## 3. Results

### 3.1. TLR4 Deficiency Prevents IH-Induced Fat Tissue Inflammation and Remodeling

**Figure 1:**
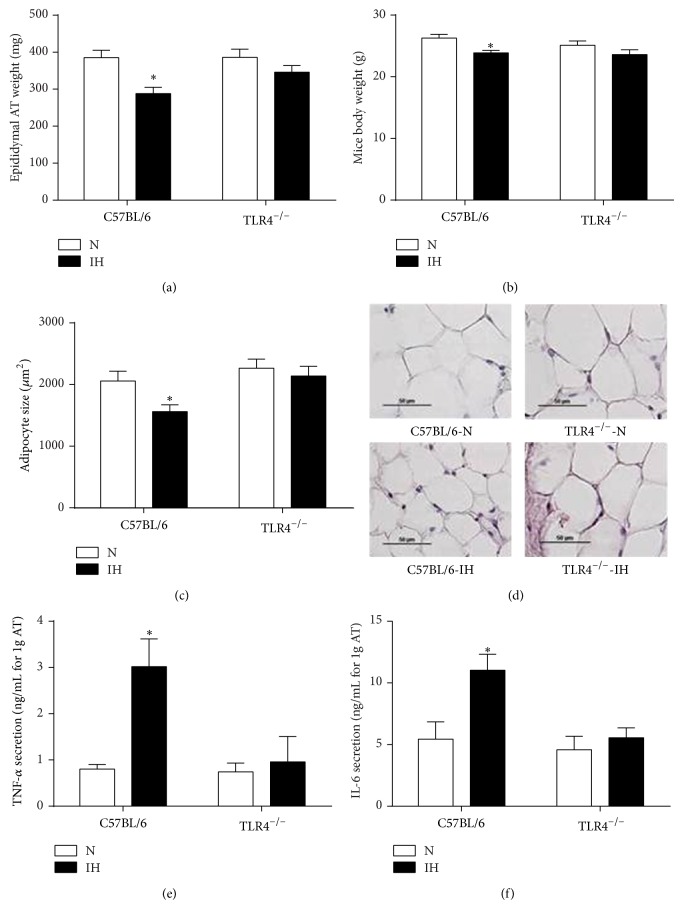
TLR4 signaling is involved in IH-induced epididymal fat alterations. Morphological and inflammatory changes of epididymal fat pads were studied in C57BL/6 and TLR4^−/−^ mice exposed to intermittent hypoxia (IH) or normoxia (N) for 4 weeks. (a) Measurements of weight of bilateral epididymal fat pads (*n* = 13–15 per group), (b) mice body weights (*n* = 13–15 per group), (c) adipocyte size (*n* = 4–6 per group), and (d) representative photographs of adipose tissue remodeling. Inflammation was studied through the release of TNF-*α* (e) and IL-6 (f) (*n* = 6–8 per group). ^*^ *P* < 0.05 IH/C57BL/6 versus N/C57BL/6.

C57BL/6 mice exposed to IH had morphological and functional alterations of EWAT. They had smaller fat pads with smaller adipocytes (Figures 1(a), 1(c), and 1(d)), and EWAT released more TNF-*α* and IL-6 compared to normoxic controls (Figures 1(e) and 1(f)). All these alterations were absent in hypoxic TLR4^−/−^ mice. Regarding body weight alterations, normoxic and hypoxic TLR4^−/−^ mice were not different from their respective control animals (Figure 1(b)).

### 3.2. TLR4 Deficiency Prevents IH-Induced Insulin Resistance

**Figure 2:**
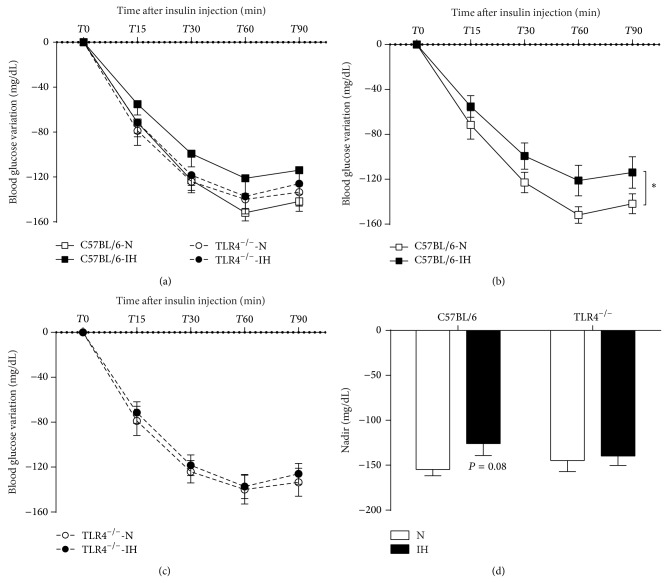
TLR4 signaling is involved in IH-induced insulin resistance. Glucose variation during the 90 minutes of the intraperitoneal insulin tolerance test (IpITT) in C57BL/6 or TLR4^−/−^ mice exposed to 1 week of intermittent hypoxia (IH) or normoxia (N) (a). IpITT presented separately for C57BL/6 (b) and TLR4^−/−^ mice (c). For each group, lowest blood glucose level (nadir) during the 90 minutes of the IpITT (d). ^*^ *P* < 0.05 IH/C57BL/6 versus N/C57BL/6, *n* = 13–15 per group.

After one-week exposure, the 4 experimental groups were assessed for insulin tolerance test (Figure 2(a)). C57BL/6 mice exposed to IH exhibited a decreased response to insulin as shown by a lower glucose decrement (Figure 2(b)) and a trend for a smaller glucose nadir compared to their normoxic controls (Figure 2(d)). The insulin response was not affected in hypoxic TLR4^−/−^ mice: the response curve was almost superposable with those of the normoxic TLR4^−/−^ animals (Figures 2(c) and 2(d)) and not significantly different from the curve of normoxic C57BL6 animals (Figure 2(a)).

### 3.3. TLR4 Deficiency Prevents IH-Induced Inflammatory Vascular Remodeling

**Figure 3:**
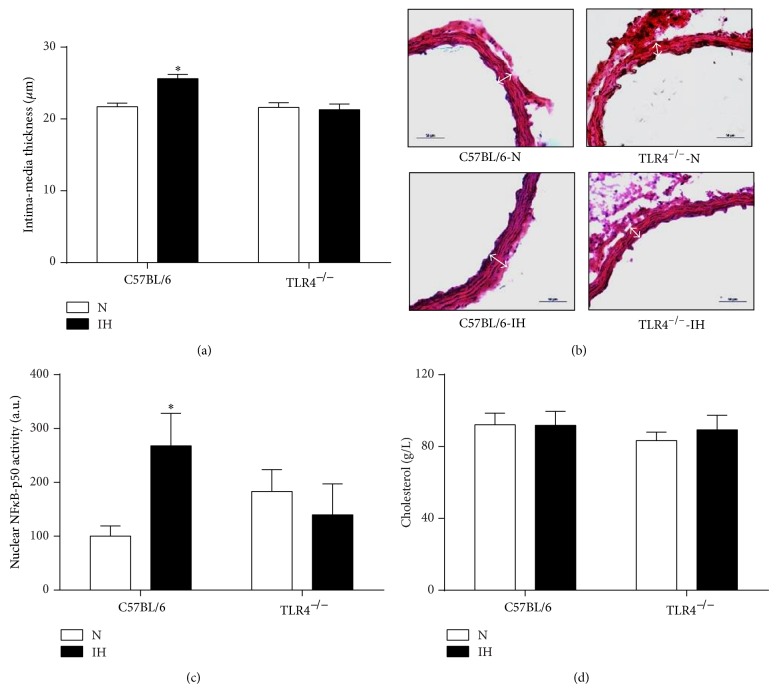
TLR4 signaling is involved in IH-induced vascular remodeling. Morphometric and inflammatory remodeling of aorta was assessed in C57BL/6 and TLR4^−/−^ mice exposed to 4 weeks of intermittent hypoxia (IH) or normoxia (N). (a) Aortic intima-media thickness quantification, (b) representative photographs of aorta remodeling (wall thickness represented by white double-headed arrows), (c) quantification of activated NF*κ*B (NF*κ*B-p50 activity) in aorta (*n* = 8–11 per group). (d) Plasma levels of total cholesterol in C57BL/6 and TLR4^−/−^ mice exposed to 4 weeks of IH or N, *n* = 12–16 per group. ^*^ *P* < 0.05 IH/C57BL/6 versus N/C57BL/6.

Hypoxic C57BL/6 mice had morphological and functional alterations of their aorta, as they exhibited larger intima-media thickness (Figures 3(a) and 3(b)) and higher NF*κ*B-p50 activity (Figure 3(c)). These alterations were not observed in hypoxic TLR4^−/−^ mice (Figures 3(a), 3(b), and 3(c)). Plasma levels of total cholesterol were not different between the 4 experimental groups (Figure 3(d)).

## 4. Discussion

The pathophysiology of OSA-induced cardiometabolic consequences is still poorly understood. There are some similarities with cardiometabolic complications due to obesity, the latter resulting from inflammation involving TLR4 signaling. Here, we showed in nonobese C57BL/6 mice that IH induced morphological and inflammatory remodeling of vascular and white adipose tissues as well as insulin resistance. These alterations were prevented in hypoxic TLR4-deficient mice suggesting that IH-induced cardiometabolic consequences involve TLR4 signaling-mediated inflammation.

### 4.1. Methodological Considerations

As previously published by our group and others, we used a deep intermittent hypoxia which mimics severe sleep apnea whereas patients mainly suffer from mild to moderate sleep apnea [7, 17, 18]. Indeed, in the absence of additional factors such as obesity, high fat diet, and genetic vulnerability, IH needs to be severe enough to induce measurable and reproducible vascular alterations, especially in C57BL/6 mice which are atheroresistant animals [7].

### 4.2. IH-Induced EWAT Remodeling Involves TLR4 Mediated Inflammation

In lean C57BL/6 mice, we showed that IH induced EWAT alterations characterized by fat pad wasting and shrunken adipocytes. These results are in agreement with previous reports in lean C57BL/6 [19] and ApoE^−/−^ [10] mice after 4 and 6 weeks of IH, respectively. Shrunken adipocytes are suggestive of lipolysis, as elevated circulating free fatty acids (FFAs) have been reported by us and others in IH-exposed animals [10, 20, 21] and in patients suffering from sleep apnea [22]. In the latter, plasma FFA levels were positively correlated with apnea-hypopnea index [22]. Activation of beta-adrenergic receptors is a well-known mechanism of lipolysis [23], and both IH and OSA are commonly associated with sympathoadrenergic activation [10, 24]. Adipose inflammation may have also contributed to lipolysis [25] as we found an increased release of the inflammatory cytokines TNF-*α* and IL6 from fat pads of hypoxic C57BL/6 mice. This is again consistent with previous reports in lean ApoE^−/−^ mice after 6 weeks of IH [10], and more recently in 3T3-L1 adipocytes exposed to fluctuating oxygen concentration [26]. These inflammatory changes may be due to local hypoxia [27], which is known to be a leading cause of EWAT dysregulation [28], as well as to systemic effects such as elevated circulating FFAs [29] and activation of the sympathoadrenergic system [10].

We found that both IH-induced morphological and inflammatory alterations of EWAT were prevented in TLR4-deficient mice. There is growing evidence from studies using murine models of obesity that activation of the proinflammatory TLR4/NF*κ*B pathway constitutes one mechanism that links inflammation and metabolic disorders [30–32]. Although TLR4 activation is known to enhance lipolysis [33, 34], we were surprised to find that EWAT wasting and adipocyte hypotrophy were completely prevented in TLR4-deficient mice suggesting that TLR4 signaling could be the main mechanism of these consequences. As observed in mice models of diet-induced obesity [30, 32], TLR4 deficiency prevented the enhanced release of TNF-*α* and IL6 in our hypoxic mice. This strengthens the role of TLR4 in IH-induced inflammation, as well as the pathophysiological similarities with obesity, that is, a normal amount of fat under hypoxia behaving like excess fat in obesity [10].

### 4.3. IH-Induced Insulin Resistance Involves TLR4 Signaling

We found that IH induced insulin resistance in C57BL/6 mice. This is in agreement with previous findings obtained in various mouse strains (genetically obese, lean C57BL/6, and ApoE^−/−^ mice) and duration of IH exposure (acute or chronic IH), using different methods to assess insulin sensitivity (HOMA-IR, ITT, hyperinsulinemic euglycemic clamp) [10, 19, 35, 36]. This confirms the role of IH in the alterations of glucose homeostasis observed in sleep apnea, the insulin resistance worsening with OSA severity, independently of obesity [37–39]. Such relationship has also been confirmed experimentally in healthy humans exposed to IH [40]. Both chronic inflammation [11] and elevated FFA levels [41, 42] are established factors causing insulin resistance in obesity. Indeed proinflammatory cytokines secreted from adipocytes are considered as a key step in obesity-induced insulin resistance, as the sole TNF-*α* neutralization in obese rats is sufficient to improve insulin sensitivity [43]. Inversely, upstream activation of the inflammatory cytokine cascade using agonists of toll-like receptors leads to insulin resistance [33, 34]. In the present study, we observed increased release of TNF-*α* and IL6 from EWAT and decreased insulin sensitivity in hypoxic C57BL6 mice. Both parameters were not impaired in TLR4-deficient mice suggesting that TLR4 mediated inflammation was involved in this metabolic consequence. Body weight alterations did not contribute to these improvements, as hypoxic C57BL/6 and TLR4-deficient mice had similar body weight.

### 4.4. IH-Induced Vascular Remodeling Involves TLR4 Signaling

We found that IH induced vascular remodeling, including morphological (larger intima-media thickness) and inflammatory changes (higher NF*κ*B-p50 activity). This confirms our previous results and others regarding the detrimental remodeling effects of IH on preatherosclerotic [17, 18] and atherosclerotic [21, 44, 45] processes. We confirmed in the present study that IH is indeed a powerful vascular stress, as only 28 days of exposure, which is very short compared to the duration of the human disease, induce early vascular alterations in the atheroresistant C57BL/6 mouse strain. Besides the well-known role of the sympathoadrenergic system and the related hemodynamic alterations [18], accumulating evidence suggests that inflammation is involved early in the pathophysiology of IH-related atherosclerosis [45]. Regarding the aggravation of atherosclerosis by IH in ApoE-deficient mice, we recently demonstrated that this deleterious effect involved inflammatory alterations of EWAT, as EWAT lipectomy prevented the proatherogenic effect of IH [10]. Interestingly, this beneficial effect occurred while insulin resistance (a well-known risk factor for atherosclerosis) was not improved by EWAT lipectomy, suggesting that EWAT inflammation could be the main determinant of IH-induced atherogenicity. Here, we showed that EWAT from hypoxic C57BL/6 mice released higher amounts of inflammatory cytokines and that this effect, as well as the morphological and inflammatory changes of aorta, was prevented in TLR4-deficient animals. Given the role of TLR4 in adipose tissue inflammation and insulin resistance [46], and the relationship between adipose tissue inflammation, insulin resistance, and vascular dysfunction [12, 47], the beneficial effect of TLR4 deficiency on arterial remodeling could be explained by the prevention of adipose inflammation and insulin resistance. Here, the role of EWAT inflammation seems to be predominant compared to the light improvement of insulin response and the absence of cholesterol alterations in these animals. IH-induced dyslipidemic alterations are indeed inconstant in mice [45, 48–50], suggesting that dyslipidemia contributes only in part to the first steps of vascular remodeling in this model. Reduced IH-driven hemodynamic alterations could be a further explanation, as TLR4-deficient mice are less susceptible to hypertension [51, 52]. Finally, a direct effect on the vascular wall is also possible, as TLR4 has been evidenced in human [53] and murine [54] atherosclerotic plaques, and inhibition of TLR4 signaling pathway attenuated diet-induced atherosclerosis in ApoE^−/−^ mice [16, 55]. A recent study also identified TLR4 signaling pathway as a direct key mediator of vascular inflammation and impairment of endothelial insulin signaling in the setting of obesity [15]. The direct effect on vasculature could even be predominant, as TLR4 deficiency prevented atherosclerosis in LDLR^−/−^ mice, with no effect on adipose tissue inflammation and whole-body insulin sensitivity [56].

## 5. Conclusion

We showed in nonobese C57BL/6 mice that IH induced morphological and inflammatory remodeling of aorta and epididymal white adipose tissue, as well as insulin resistance. These alterations were prevented in TLR4-deficient mice suggesting that IH-induced cardiometabolic consequences involved inflammation mediated by TLR4 signaling. The precise mechanisms and the specific role of one type of tissue or cell (e.g., adipose tissue) remain to be determined as TLR4 knockout used in the study was not cell specific. TLR4 is indeed expressed on many cell types, predominantly those of the immune system, but also on nonhematopoietic cell types (e.g., endothelial, epithelial cells, etc.). Despite these limitations, as a practical point of view, the whole body is exposed to hypoxia during sleep apnea, and TLR4 in various cells is likely to be involved in the numerous complications of sleep apnea. Moreover the available treatment that blocks TLR4 activation (eritoran) is not cell specific. Whether our results can be extrapolated to the human disease remains to be determined. However, one clinical study has recently investigated the activation of TLR4 signaling pathway in OSA. The authors found increases in TLR4 expression, NF*κ*B nuclear binding, and release of IFN*γ*, TNF-*α*, and IL-6 in circulating monocytes [57]. There are therefore similarities between these clinical findings and our experimental results suggesting that targeting TLR4/NF*κ*B pathway could provide further therapeutic options for sleep apnea patients.
